# Formulating Mechanically Robust Composite Restorative Materials for High Performance

**DOI:** 10.3390/jfb16030101

**Published:** 2025-03-13

**Authors:** Austyn Salazar, Natalie Anderson, Jeffrey Stansbury

**Affiliations:** 1 Craniofacial Biology, School of Dental Medicine, University of Colorado Anschutz Medical Campus, Aurora, CO 80045, USA; austyn.salazar@cuanschutz.edu (A.S.); natalie.j.anderson@cuanschutz.edu (N.A.); 2 Chemical and Biological Engineering, University of Colorado, Boulder, CO 80303, USA

**Keywords:** dental composite restorative, photopolymerization, (meth)acrylate resin

## Abstract

Although dental resin composite restoratives offer a widely used direct-placement treatment option aimed at replacing the form and function of a natural tooth, there are several clinically relevant performance aspects of these materials that can be improved. The formulation of the resin matrix phase of dental composites for high-efficiency photopolymerization leading to polymers with excellent mechanical properties has always been a challenge that is addressed here through the use of structurally new and more reactive monomers as well as the formation of polymer networks that incorporate non-covalent reinforcing interactions. The purpose of this study was to validate that a set of tetraurethane diacrylates (TUDAs) with a novel configuration of their urethane linkages in coordination with acidic comonomers could be devised to obtain highly robust new composite materials. Due to the novel molecular design, this exploratory approach was conducted using reaction kinetics and three-point bend testing to assess the performance. Conversion and mechanical properties were measured to refine these formulations prior to the addition of filler. The initial formulations demonstrated outstanding dry mechanical test results that subsequently showed a major intolerance to water storage, which led to a model study using urethane diacrylate (UDA) followed by the addition of hydrophobic TUDA monomers. Once the resin formulations were optimized, silane-treated particulate filler was added to determine the effectiveness as composite materials. The final formulation used a hydrophobic, aromatic TUDA along with 4-methacryloxyethyl trimellitic anhydride (4-META) as a latent acidic comonomer and a mixture of acrylic acid (AA) and methacrylic acid (MAA). This formulation achieves a very high level of both reactivity and mechanical properties relative to current dental composite restoratives.

## 1. Introduction

Resin-based composites (RBCs) have been widely used in dental restorative treatment due to their good esthetics and suitability toward rapid intraoral polymerization that results in photopolymers with good mechanical properties [1]. The switch from amalgam to RBCs has been dramatic; however, direct RBC restorations placed in posterior teeth have almost double the failure rate of amalgam restorations [2]. Stronger, stiffer, but simultaneously tougher composites are needed since in dental restorations with three or more restored surfaces, failure by composite fracture is two times more prevalent than secondary caries as a failure mode [3,4]. RBCs rely on the cooperative mechanical properties of both the glass filler and the polymer matrix. The challenge taken on here is the development of an RBC material with improved mechanical property capabilities while also attaining good photo-reactivity and high conversion to limit potential leaching and biocompatibility issues [5,6,7]. Glass filler enhances the modulus, hardness, and wear resistance of the composite to approximate as closely as possible the character of a natural tooth [8]. The polymer matrix relies on the fluidity of the resin in its precure state and the properties of the near-ambient restricted reaction to the polymer activated by brief exposure to visible light [9]. For this reason, it is crucial to design and formulate higher-performance resins that can potentially offer extended clinical service life, which is the eventual goal here.

Bisphenol A-glycidyl methacrylate (BisGMA) and triethylene glycol dimethacrylate (TEGDMA) were originally used and continue to be used in RBCs because of their relatively good mechanical properties. BisGMA has a stiff aromatic core along with secondary hydroxyl groups that act as hydrogen bond donors and acceptors. These characteristics are advantages, but a drawback is that monomeric BisGMA is extremely viscous (~1200 Pa*s) [10]. TEGDMA serves well in that role to reduce viscosity and raise the attainable degree of conversion, allowing a practical resin formulation, but it also compromises the favorable non-covalent interactions. The positive polymer reinforcement due to hydrogen bonding present with BisGMA also led to the use of urethane dimethacrylate (UDMA) as a possible additive or alternative primary monomer. UDMA has a far lower viscosity of 8.9 Pa*s, and the non-covalent network provided by the urethane group interactions enhances polymer mechanical properties [11]. UDMA has been used in a multitude of different RBCs, and many variations of urethane-based monomers have been used primarily in experimental work [12,13]. Comparing the degree of conversion and other kinetic properties as well as the glass transition temperature (Tg) of homopolymers of UDMA, TEGDMA, BisGMA, and BisEMA was the primary focus, emphasizing the role that mobility in a system plays in determining the overall Tg. Additionally, novel urethane-based monomers have been implemented in composite work. An example of urethane variations is the reaction of 1,3-bis(1-isocyanato-1-methylethyl) benzene (MEBDI) with several hydroxyalkyl methacrylates [12]. In the comparison of polymer properties to that of UDMA/TEGDMA or BisGMA/TEGDMA, the MEBDI-based monomers showed a general trend of higher flexural strength and modulus. The potential for improvement in mechanical properties as well as examples of urethane monomers with inherently high reactivity [14] provides further justification to pursue the design and development of novel urethane-based monomers for dental applications.

A prior study was conducted comparing the benefits of including UDMA in formulations consisting of BisGMA, BisEMA, and TEGDMA [15]. The effect of hydrogen bonding is evident through the mechanical properties of the compositions tested. Although statistical differences between samples were not shown, flexural strength tends to increase with an increase in UDMA in formulations containing BisGMA or BisEMA. Separately, the introduction of a methacrylic acid (MAA) with UDMA showed promising results with MAA acting as a very effective reactive diluent in addition to producing enhanced mechanical properties compared with a BisGMA/UDMA/TEGDMA control [16,17]. The formulation of higher-performing resins has been a challenge, and the approach taken here utilizes the urethane–acid non-covalent interactions but expands this to include novel tetraurethane diacrylate (TUDA) monomers, which introduce pairs of relatively close-spaced urethane linkages. These monomers were formulated with (meth)acrylic acid (MAA/AA) in an attempt to optimize photo-reactivity and conversion of the resin as a means to limit leachable components and potential biocompatibility concerns that are typically more pronounced with acrylate-based materials [18,19]. In a different approach, oligomeric urethanes that included carboxylic acid side chains produced interesting results when used as additives in a dental materials study [20]. The use of MAA/AA in the current formulations provides a good mechanical performance, but further investigation of these novel urethane monomers with other acidic comonomers is likely needed. Small molecule monomers can greatly impact biocompatibility and polymerization shrinkage as well as raise concerns with volatility and odor in the precure state. While these are all important considerations, the purpose of this preliminary investigation was to validate that novel acid-reinforced TUDA-based formulations could be designed to provide highly robust RBCs. The results here are intended to serve as a foundation for subsequent refinement work on the resin formulations, including the selection of alternate acidic monomers, as well as the assessment of the clinically relevant properties in more comprehensive detail [21,22].

For any anterior restoration, the optical properties of composite materials are an essential element for consideration. These esthetic-related properties include color and color stability, translucency in the pre-/post-cure state, opalescence, gloss retention, and masking ability [23,24]. With the current acid-reinforced urethane (meth)acrylate resins, the prospect of high polymeric modulus has the potential to limit wear and surface roughness, which can promote better gloss retention, while high degrees of conversion have been associated with better color stability [25]. The resin refractive index can be adjusted with both the acid and the urethane monomeric components to control composite translucency. The inclusion of acid-functional monomers may raise some concerns over color stability. However, a prior study of accelerated aging with acid-containing self-etch composites showed that strong acids promoted color change but that milder acids such as those derived from the 4-methacryloxyethyl trimellitic anhydride (4-META), as used here, did not have the same effect [26].

## 2. Materials and Methods

Ethylene carbonate (EC), methacrylic acid (MAA), acrylic acid (AA), isophorone diamine (IPDA), and 2,2-dimethoxy-2-phenylacetophenone (DMPA) were purchased through Sigma-Aldrich, St. Lewis, MO, USA. Trimethyl hexamethylenediamine (2,2,4- and 2,4,4- mixture) (TMHDA), xylylene diamine (XDA), and dibutyltin dilaurate (DBTDL) were purchased through TCI, Tokyo, Japan. 2-Isocyanatoethyl acrylate (IEA) and 2-isocyantoethyl methacrylate (IEM) were generously provided by Nagase, Osaka, Japan. Dichloromethane (DCM) was purchased through Fisher Scientific, Waltham, MA, USA. 4-META and mono-2-(methacryloyloxy)ethyl phthalate (MEP) were purchased from Esstech, Essington, PA, USA. Urethane diacrylate (UDA) and dimer diisocyanate urethane dimethacrylate (DDI-HEMA) were purchased from Designer Molecules, San Diego, CA, USA. Particulate barium glass filler (average 0.7 μm) was purchased from Schott, Mainz, Germany, and treated with methacryloyloxypropyl trimethoxysilane that was purchased from Sigma-Aldrich via a previously described method [27,28]. Isostearyl methacrylate (ISMA) was purchased from Kowa, Nagoya, Japan.

**Figure 1:**
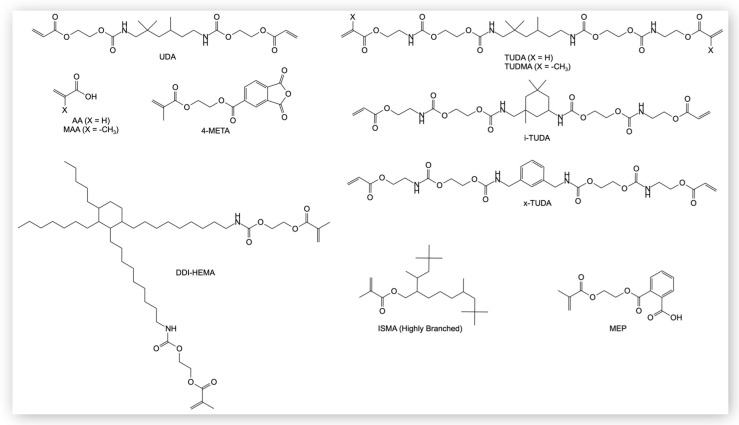
Structures of commercially and synthetically obtained monomers used in this study.

Synthesis of Tetraurethane Monomers:

In a 250 mL round bottom flask, 2.0 g of EC (22.7 mmol) was dissolved in 20 mL of DCM. A total of 1.8 g of TMHDA (11.35 mmol) was dissolved in 20 mL of DCM and cooled to 4 °C. The round bottom with EC was put in an ice bath and an addition funnel was set up to add the TMHDA dropwise. With the amine added, an additional 10 mL of cold DCM was introduced, and the reaction was allowed to warm up to 25 °C. The reaction was monitored by the 1800 cm^−1^ cyclic carbonate peak in the mid-IR, and it was assumed complete once this peak disappeared (~24 h). To the same flask, 3.2 g of IEA diluted with 20 mL of DCM was added dropwise to the reaction along with 0.01 wt% of DBTDL as a catalyst. The disappearance of the isocyanate peak at 2270 cm^−1^ was used to determine when the second reaction step was complete. The solvent was removed via a rotary evaporator under reduced pressure, and the TUDA monomer (Figure 1) was used without further purification. Analogous reactions were conducted with IPDA and XDA serving as the diamine. The TUDA monomers using TMHDA and IPDA were both clear, highly viscous liquids, and the TUDA monomer using XDA was isolated as a white solid (mp = 92 °C). Tetraurethane dimethacrylate (TUDMA) was synthesized with the same protocol using TMHDA and EC but used IEM instead of IEA. The TUDMA was also obtained as a clear, viscous liquid. Yield varied between 90 and 99% for these reaction schemes.

Sample formulation: Initial formulations were based on complementing acidic moieties with urethane moieties, and so resins were formulated based on equivalents of acidic functionality to urethane functionality within the comonomers along with 0.1 wt% of DMPA as a UV-based photoinitiator for the preliminary studies. DDI-HEMA was added as a hydrophobic urethane comonomer using either an 80:20 or 90:10 TUDA:DDI-HEMA molar equivalent. Alternatively, a highly branched isostearyl methacrylate (ISMA) was added at 10 wt% of the total formulation. Composites were formulated using 60 wt% of barium glass that was incorporated into the resin with a SpeedMixer (FlackTek, Landrum, SC, USA) in incremental additions using 1500 rpm in 30 s bursts until homogeneous.

Sample preparation: Samples for kinetics (n = 3) and 3-point bending (n = 7) were prepared, respectively, by filling a 1 × 10 mm circular mold or a 2 × 2 × 25 mm mold that was placed between mylar film and glass plates. All flexural samples were photocured under an Acticure 4000 (EFOS, Scotia, NY, USA) spot curing lamp using a 365 ± 10 nm filter and a 100 mW/cm^2^ incident irradiance at 2 min per side. Kinetic samples were photocured using the same lamp and filter, but the intensity was reduced to either 30 mW/cm^2^ or 10 mW/cm^2^.

Conversion: A Nicolet 6700 FTIR (Thermo Nicolet, Waltham, MA, USA) was used to measure the double bond conversion of each sample in the near-IR region in transmission mode. The 6150 cm^−1^ =CH_2_ peak area was integrated before and after curing, and the formula below was used to calculate conversion:

Conversion = 1 − (Area_Initial_/Area_Cured_)

Mechanical testing: A Mechanical Testing System (MTS) was used to analyze flexural samples under 3-point bending. The crosshead speed was 1 mm/min with a span length of 20 mm. Wet samples were subject to 48 h in a 37 °C water bath and tested immediately after removal from the bath.

## 3. Results and Discussion

**Figure 2:**
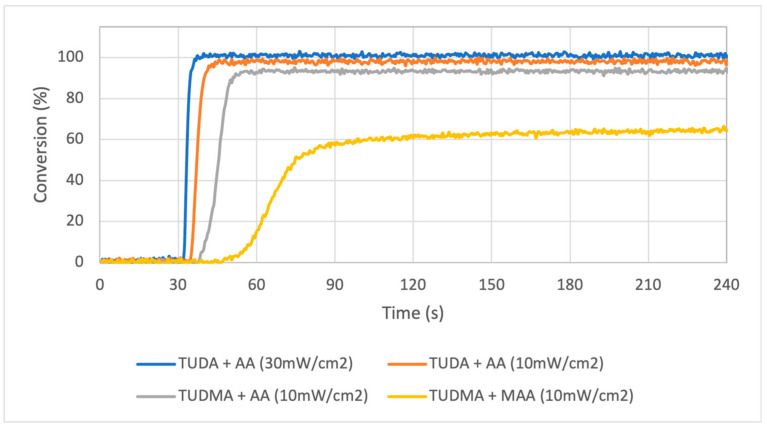
Conversion profiles of TUDA + AA, TUDMA + AA, and TUDMA + MAA at a 365 nm irradiance of either 30 mW/cm^2^ or 10 mW/cm^2^. Each formulation contained 8 equivalents of AA and 1 equivalent of urethane monomer. Irradiance was continuous beginning at 30 s.

The initial attraction of the TUDA monomer was the rapid and near-complete reactivity during ambient photopolymerization with AA. In Figure 2, the low irradiance conversion profiles of TUDA/TUDMA with AA/MAA were measured to allow a meaningful differentiation of the respective reaction rates. The initial formulation of one equivalent of TUDA with four equivalents of AA was run at an irradiance of 30 mW/cm^2^, but even with this limited light intensity, the formulation cures at such an accelerated rate that the irradiance was further lowered to 10 mW/cm^2^. Under these conditions, the all-acrylate formulation (TUDA + AA) was able to reach >98% within 12 s of the lamp being turned on; however, substituting TUDA with TUDMA modestly decreased the limiting conversion as well as delayed the reaction onset and time to vitrification. The most dramatic difference can be seen with the TUDMA/MAA combination, which resulted in a substantial decrease in the overall reaction rate and conversion as well as a much more gradual transition to a slowly increasing plateau. For comparison, a UDMA/MAA ambient photocure reaction results in a conversion of 64.8 ± 1.5%, which is equivalent to that obtained with TUDMA/MAA. In terms of resin photo-reactivity, AA is a key component in these formulations allowing for much higher reaction rates along with extremely high degrees of conversion.

**Figure 3:**
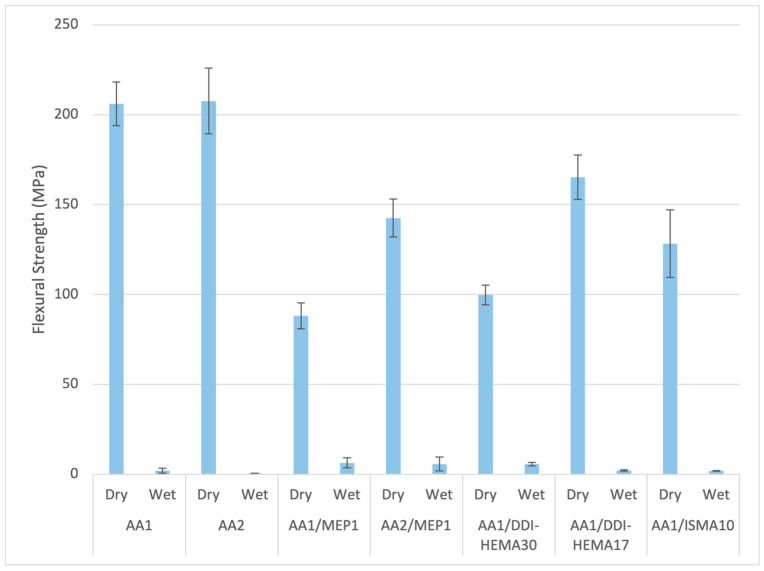
Flexural strength of formulations using TUDA/AA with other relatively hydrophobic monomers. Numbers with either AA or MEP comonomer show the equivalent of that acid comonomer relative to one equivalent of TUDA. Hydrophobic monomers (ISMA or DDI-HEMA) use a number corresponding to weight percent of the monomer in the total formulation. The dry vs. wet property results are statistically different (*p* < 0.001) in every case.

**Figure 4:**
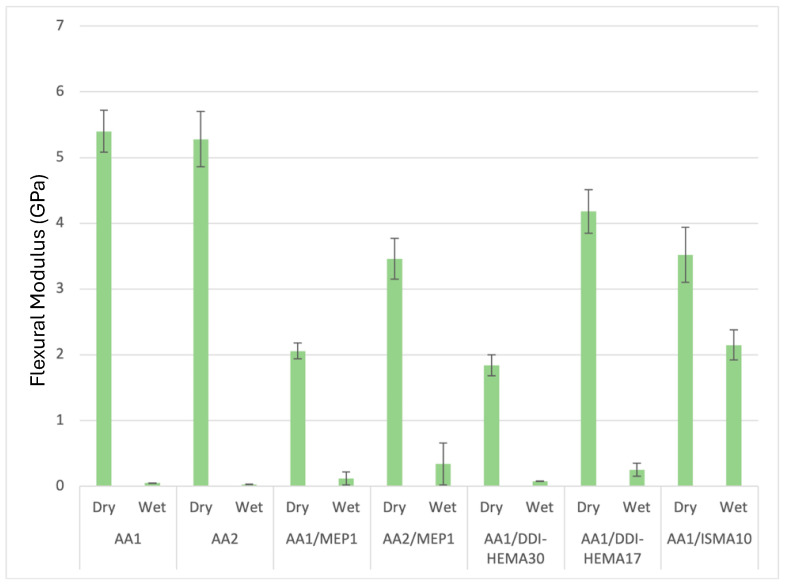
Flexural modulus of formulations using TUDA/AA with other relatively hydrophobic monomers. Numbers with either AA or MEP comonomer show the equivalent of that acid comonomer relative to one equivalent of TUDA. Hydrophobic monomers (ISMA or DDI-HEMA) use a number corresponding to weight percent of the monomer in the total formulation. The dry vs. wet property results are statistically different (*p* < 0.001) in every case.

Non-covalent interactions within the TUDA crosslinked network provide physical reinforcement that enhances the mechanical properties of the dry polymers. As seen in Figure 3 and Figure 4, as a UDMA analog, the TUDA monomer with AA shows great potential exceeding 200 MPa flexural strength and the 5 GPa flexural modulus as the ambient photocured unfilled polymers. These mechanical properties of the TUDA/AA photopolymer are substantially greater than that of UDMA/MAA, which provides a flexural modulus of 1.54 ± 0.05 GPa and a flexural strength of 124.2 ± 10.4 MPa. This superiority in both mechanical properties and degree of conversion makes the TUDA/AA composition an interesting option for potential use in ambient cure RBC applications. Since these formulations are dependent on an acidic comonomer like AA or MAA, it is reasonable to assume that they may not maintain this degree of strength and stiffness in a wet environment [29]. The results of the effect of water on strength in Figure 3 and modulus in Figure 4 are profoundly evident. The dry mechanical properties along with the high conversion offer an interesting foundation for an RBC, but obviously, the wet property degradation needs to be resolved for these to be practically considered. Poly(AA) is notably hydrophilic, particularly in an alkaline state, but its water uptake is an inherent concern even under neutral conditions [30]. In an attempt to moderate the drop in wet polymer properties, hydrophobic comonomers were added into these formulations. The aromatic MEP acidic monomer was added in combination with AA to determine if this more hydrophobic resin modification would provide benefits. MEP addition proportionally reduces both the dry strength and stiffness relative to the TUDA/AA formulation while providing limited rescue of the wet properties. DDI-HEMA was introduced at 10–20 mol% with TUDA because it is an extremely hydrophobic cycloaliphatic diurethane monomer [31], but it too produced decreased dry strength and stiffness with only modest benefit to the wet results. The branched aliphatic ISMA does not contain urethane functionality, but when used in urethane-based formulations, it was found to increase overall hydrophobicity with limited potential for phase separation. While the 10 wt% ISMA addition to TUDA/AA gave the highest wet modulus result in this series, it also decreased the dry mechanical properties and failed to promote any improvement in wet strength. Figure 3 and Figure 4 clearly show a drop in dry flexural strength and modulus with these added comonomers to about 150 MPa and 3.5 GPa, respectively. Most critically, this is occurring without producing the necessary retention of the wet polymer strength results.

**Figure 5:**
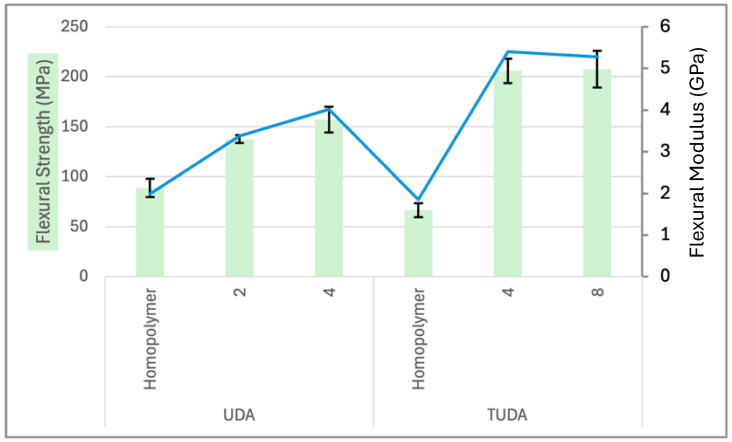
Flexural strength (bars, left axis) and modulus (line, right axis) of formulations consisting of either UDA or TUDA as homopolymers or copolymers with AA. Formulations are denoted as homopolymers or copolymers with the number of equivalents of AA per urethane monomer.

UDA was considered here as an analog of TUDA that could also offer enhanced cure rates and elevated conversion. To better understand the similarities and differences between UDA and TUDA, these two urethane acrylates were compared as homopolymers and as copolymers with AA under ambient photocure and dry storage conditions. As a homopolymer, UDA exhibits a slightly higher modulus and significantly increased flexural strength relative to TUDA. This may be a result of preferential intramolecular hydrogen bonding interactions between the closely spaced urethane groups in TUDA that limit intermolecular engagement that contributes to the reinforcement of the polymer network. In addition, the TUDA crosslink spacer length is significantly greater than that in UDA, which reduces the covalent network density. Taking this further, since TUDA has four urethane groups, the effect of introducing an equivalent acid-to-urethane ratio is amplified, including a further reduction in the final crosslink density of the TUDA/AA versus UDA/AA polymers. In Figure 5, an increase in AA with UDA from 0 to 2 equivalents and from 2 to 4 equivalents shows very positive enhancement in flexural strength and modulus. As further justification to continue the development of acid-reinforced tetraurethanes, the TUDA/AA copolymers demonstrate a tremendous increase in flexural strength from 67 MPa for the TUDA homopolymer to 206 MPa for the copolymer both with four and eight equivalents of AA, and a comparable dramatic increase was seen in the modulus results as well. In this case, further incorporation of AA had no effect on the polymeric mechanical properties. These results clearly indicate that the urethane–acid hydrogen bonding enhances both the strength and the stiffness of UDA-based copolymers, but by increasing the number of urethane groups and lowering the crosslink density in TUDA-based formulations, substantially greater improvements in polymeric properties can be achieved. Following this, we conducted a model dry/wet storage study with UDA and various combinations of acidic comonomers with the goal of identifying comonomers for use with TUDA to not only enhance dry polymer properties but also to mitigate wet property decline.

**Figure 6:**
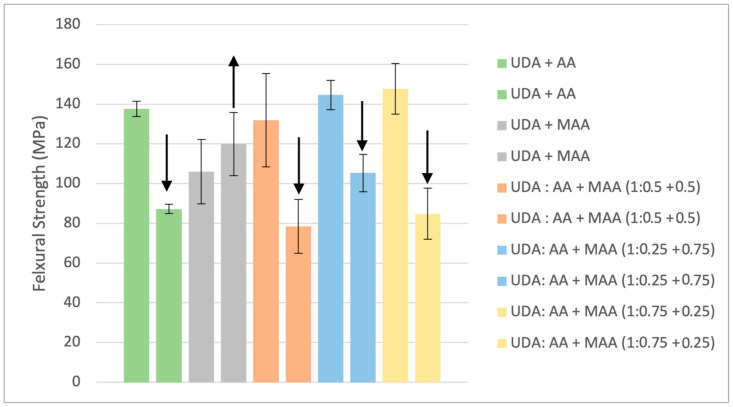
Flexural strength of formulations using UDA, AA, and MAA. Arrows highlight the direction of the change in the wet property relative to dry testing. UDA was formulated with different equivalents of AA and MAA. The increase in wet strength for the UDA/MAA polymer was not statistically significant (*p* = 0.13), but the decreases in wet strength noted for all other formulations were significant (*p* < 0.001).

**Figure 7:**
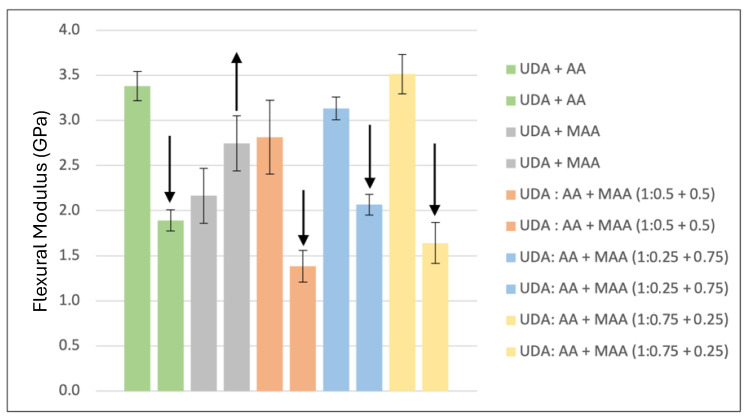
Flexural modulus of formulations using UDA, AA, and MAA. Arrows highlight the direction of the change in the wet property relative to dry testing. UDA was formulated with different equivalents of AA and MAA. The increase in wet modulus for the UDA/MAA polymer was statistically significant (*p* = 0.004), and the decreases in wet modulus noted for all other formulations were also significant (*p* < 0.001).

While UDA does not have the same potential for acid-reinforced polymer mechanical property improvement as TUDA, it is also evident in Figure 6 and Figure 7 that the effect of water on UDA/AA copolymers, while still significant, is much less dramatic than that seen with TUDA/AA polymers. Better water tolerance would be expected due to the overall greater hydrophobicity and crosslink density of UDA/AA compared with TUDA/AA. Even though MAA is structurally similar to AA, the additional methyl group in MAA makes it less hydrophilic, and when substituted for AA in UDA copolymers, it reduces the dry strength and modulus compared with UDA/AA, possibly due to the lower degree of conversion attained with the MAA-containing resins (Table 1). However, the use of MMA in place of AA retains or even improves the wet mechanical properties relative to the dry results. Therefore, this model study also evaluated mixtures of the AA and MAA comonomers with UDA as a possible route to balance the positive influence of AA on reactivity and final conversion, with its deleterious effects on wet mechanical properties. Based on the flexural strength and modulus (Figure 6 and Figure 7) as well as the degree of conversion (Table 1), the preferred ratio of the reactive diluents with UDA is 0.25:0.75 AA:MAA (blue bars) due to reasonably high dry mechanical properties that suffered the least percentage drop upon water storage along with considerably higher ambient cure conversion compared with MAA alone. Considering this acid–urethane monomer composition gave the best balance of conversion and dry/wet mechanical properties, it was used going forward with TUDA and related tetraurethane derivatives.

**Figure 8:**
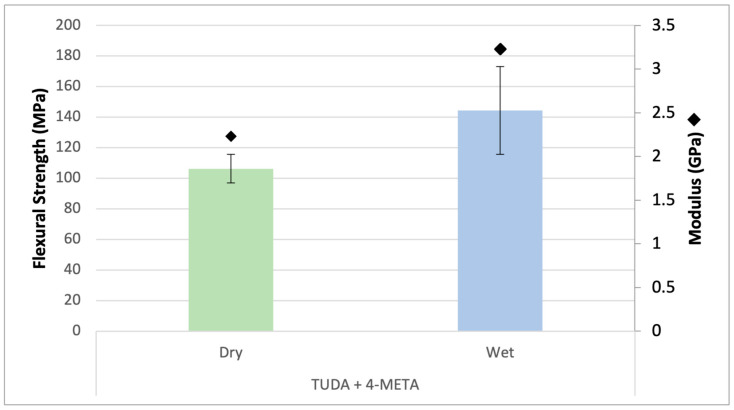
Flexural strength and flexural modulus of TUDA and 4-META. Flexural strength (bars) and flexural modulus (diamond) show an increase in properties under wet conditions.

Since MEP, DDI-HEMA, and ISMA as comonomers with TUDA all resulted in compromised dry mechanical properties and provided limited support under wet conditions, another alternative, 4-META, as a potential latent diacid with a hydrophobic aromatic group, was also evaluated as a comonomer in urethane resins. 4-META was used at a 2:1 ratio with TUDA in dry and wet conditions, and as anticipated, the photopolymer from this formulation underperformed mechanically in the dry state in which the reinforcing acidic groups remained undeployed. However, after exposure to water, the cyclic anhydride appears to be efficiently opened as indicated by loss of the strained carbonyl absorption >1800 cm^−1^ in the mid-IR. The benefit of the diacid presentation was that it not only accommodated water, but the wet properties significantly improved over the dry state. Figure 8 shows an increase in flexural strength from 125 to 140 MPa and an increase in modulus from 2.25 GPa to 3.25 GPa with water storage. Since these formulations rely on physical network reinforcement via hydrogen bonding between the acid and urethane groups, this combination raises the possibility of an integrated diacid in coordination with the close-space urethanes of TUDA. The 4-META would also create a more hydrophobic local environment for the acid–urethane hydrogen bond compared with AA.

**Figure 9:**
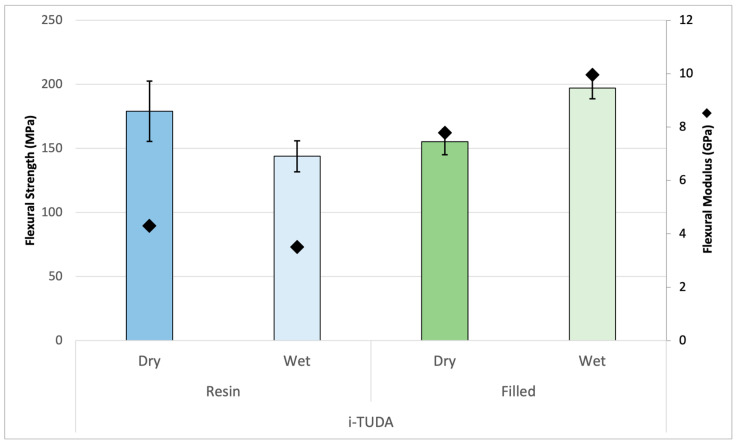
Polymers from resin formulation composed of i-TUDA, AA, and 4-META unfilled (blue bars) and with silanized filler (60 wt%, green bars) under dry/wet storage conditions. The statistically significant (*p* < 0.003) decrease in the unfilled flexural strength (bars) and modulus (diamond) under wet conditions is countered by a significant increase (*p* < 0.001) in these same properties in the filled analog materials.

**Figure 10:**
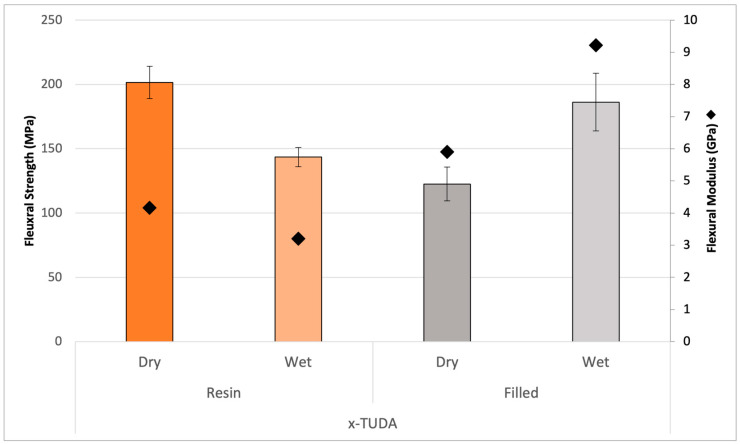
Polymers from resin formulation composed of x-TUDA, AA, and 4-META unfilled (orange bars) and with silanized filler (60 wt%, gray bars) under dry/wet storage conditions. The statistically significant (*p* < 0.001) decrease in the unfilled flexural strength (bars) and modulus (diamond) under wet conditions is countered by a significant increase (*p* < 0.001) in these same properties in the filled analog materials.

Since 4-META, albeit as a crystalline solid that limits its diluent character, was successful as a comonomer to promote the wet property results of the TUDA polymer, it was added into formulations containing AA, which is a very effective reactive diluent for urethanes, and the i-TUDA monomer. Figure 9 shows the i-TUDA monomer with AA and 4-META at a ratio of 3:1 compared to what was previously tested. At this elevated ratio, any effects of the water should be magnified due to the higher acid content. In Figure 9, the polymerized unfilled resin is measured both dry and wet, which shows a decrease in flexural strength from 181 MPa to 144 MPa, which represents a great improvement from the wet property results of the initial TUDA/AA formulation. This relatively modest drop is likely the result of a reasonable balance between the negative wet effect of AA versus the positive influence of the 4-META upon water exposure. Additionally, this resin was combined with silane-treated 0.7 μm barium glass filler (60 wt% filler) and tested both dry and wet. This showed a similar trend to that of TUDA/4-META where both the ambient photopolymerized composite stiffness and strength increase in the presence of water. Unlike the analogous resin sample, in the filled material, the divergent wet effects of AA and 4-META appear to favor the latter, and the polymer stiffens and strengthens significantly when wet. Figure 9 shows this same trend of modulus increase for the composite in water although both the dry and wet results show the expected modulus increase compared with the unfilled resin. Figure 10 shows very similar trends, but in this case, using the x-TUDA monomer with its aromatic core structure. The photocured x-TUDA and i-TUDA composite formulations are very comparable with the wet flexural strength results at nearly 200 MPa, which is remarkable considering the initial TUDA/AA resin entry point. With the utilization of glass filler, the refractive index (RI) plays a role in the depth of cure potential. In addition to the expected increase in hydrophobicity in proximity to the urethane groups, the higher refractive index (RI) of the aromatic x-TUDA in combination with the 4-META was designed to offer a better match with the barium glass filler. The RI of the resin formulations represented in Figure 9 and Figure 10, containing i-TUDA, is 1.515, whereas the x-TUDA formulation reached an RI of 1.530, which is very well matched to the specific glass filler used here and may allow for better esthetics and a greater depth of cure in photocured composite applications [32,33].

The culmination of using 4-META as a comonomer with TUDA, the acid–urethane model study with UDA, and the decision to use x-TUDA led to the development of a modified tetraurethane-based formulation for potential use as a dental resin composite. This optimized formulation was made up of 1 mole of x-TUDA, 1 mole of AA, 3 moles of MAA, and 2 moles of 4-META. Table 2 shows the final properties of this formulation as an unfilled resin. The conversion was not nearly as high as the initial TUDA/AA formulation (>95%), likely due to the overall ratio of methacrylate to acrylate reactive groups. Under ambient conditions, acrylates tend to reach higher conversion, and with the addition of more MAA and 4-META, the contribution of the acrylate groups is significantly diminished. As a comparison for the mechanical property results obtained here, a recent review identifies a flexural strength range of 76-132 MPa for packable dental composites [34] and our own testing of the popular 3M Filtek Universal composite (Solventum, St. Paul, MN, USA), which also contains UDMA, provided a dry flexural strength of 152.2 ± 14.4 MPa that under wet storage decreased to 132.8 ± 3.3 MPA, which constitutes a statistically significant decline (*p* = 0.01).

## 4. Conclusions

Due to the non-covalent interactions of urethane–acid chemistry and based on previous research, a dental resin was formulated to meet or exceed the performance of other RBCs but also to retain or increase their mechanical properties when in the presence of water. The beneficial polymer network reinforcing effect arising from the hydrogen bonding between the urethane and acid functional groups in (meth)acrylates has previously received little attention. We demonstrated the use of this approach as well as introduced a series of novel tetraurethane monomers that offer further advances in the performance level in comparison to conventional diurethane-based photopolymers. Initial formulations achieved the unusual combination of near quantitative ambient conversion along with exceptional polymer modulus and strength results. However, the detrimental effect of water on the mechanical properties of formulations with excessive AA is dramatic and would automatically eliminate these materials from further consideration for RBC applications. Hydrophobic monomers were employed to reduce this outcome with very limited success, and so, a model study using UDA as an analog to TUDA enabled a better decision in the formulation of the resin using a mixture of MAA and AA. 4-META was investigated as another hydrophobic comonomer, but one able to hydrolyze in an aqueous environment and subsequently contribute to the important non-covalent interactions. 4-META was able to not only salvage these mechanical properties as a resin but enhance them relative to the dry results when incorporated along with barium glass filler. Further evaluation of properties including polymerization shrinkage and stress as well as any exotherm during high irradiance visible light activation and cytotoxicity testing would be required before advancing these composites for broader consideration. However, this preliminary study does highlight some interesting potential for these new clustered urethane-based, BisGMA-free restorative materials.

## 5. Patents 

Stansbury JW, Salazar A. Curable Composition with Urethane (Meth)acrylate Monomer and Acidic Comonomer. Provisional application 63/463,406 filed 2 May 2023.

## Figures and Tables

**Table 1:** Formulations of UDA mixed with varied ratios of AA/MAA comonomers and the respective conversion post ambient cure. The molar equivalent of acid groups to urethane groups is shown above with corresponding ambient cure conversion. Superscript letters refer to statistically significant groupings (ANOVA and Tukey–Kramer post hoc test).

Acid Comonomer	Molar Eq. AA	Molar Eq. MAA	Conversion (%)
AA	1	0	95.8 ± 1.6 ^a^
MAA	0	1	80.8 ± 0.9 ^b^
AA + MAA	0.5	0.5	89.6 ± 3.3 ^c,d^
	0.75	0.25	93.1 ± 0.5 ^a,c^
	0.25	0.75	87.0 ± 1.4 ^d^
	1	1	92.3 ± 2.0 ^a,c^

**Table 2:** Conversion and dry/wet mechanical properties of the photopolymerized resin formulation. The increases in wet strength and modulus are not statistically significant (*p* > 0.06).

		Conversion, %	Flexural Strength, MPa	Flexural Modulus, GPa
x-TUDA + AA/MAA + 4-META	Dry	78.0 ± 1.5	175.7 ± 38.0	4.0 ± 0.6
	Wet		202.0 ± 22.0	4.3 ± 0.6

## Data Availability

The original contributions presented in this study are included in the article. Further inquiries can be directed to the corresponding author.
